# Subarachnoid hemorrhage caused by a paradoxical septic embolism from pacemaker lead infection: A case report

**DOI:** 10.1016/j.radcr.2026.10.016

**Published:** 2026-10-29

**Authors:** Takayuki Yamashiro, Hirokuni Sakima, Toshiki Yamashiro, Masashi Nakamatsu, Kenya Kusunose

**Affiliations:** a Department of Cardiovascular Medicine, Nephrology, and Neurology, Graduate School of Medicine, University of the Ryukyus, Okinawa, Japan; b Infection Control Team, University of the Ryukyus Hospital, Okinawa, Japan

**Keywords:** Paradoxical septic embolism, Subarachnoid hemorrhage, Pacemaker lead infection, Infective endocarditis

## Abstract

Subarachnoid hemorrhage resulting from a paradoxical septic embolism associated with right-sided cardiac infection has not been previously reported. An 85-year-old woman with a history of aortic valve replacement and right ventricular pacemaker implantation 7 years earlier presented with sudden-onset left upper limb weakness and a small subarachnoid hemorrhage over the right frontal convexity. Contrast-enhanced computed tomography revealed a small aneurysm in the distal right middle cerebral artery. Transthoracic echocardiography revealed vegetation on the right ventricular pacemaker lead, and blood cultures showed growth of *Listeria monocytogenes*. Transesophageal echocardiography revealed a patent foramen ovale without evidence of infection in the left cardiac chambers or bioprosthetic aortic valve. The patient was diagnosed with subarachnoid hemorrhage due to the rupture of an infectious intracranial aneurysm, presumably caused by a paradoxical septic embolism originating from the pacemaker lead vegetation. After antibiotic therapy, follow-up transthoracic echocardiography revealed complete resolution of the lead vegetation. To our knowledge, this is the first reported case of subarachnoid hemorrhage resulting from a presumed paradoxical septic embolism associated with a pacemaker lead infection.

## Introduction

Septic emboli of cardiac origin, such as those from infective endocarditis or infected pacemaker leads, can cause various neurological complications, including ischemic stroke, intracranial hemorrhage, and subarachnoid hemorrhage, due to the formation and rupture of infectious intracranial aneurysms. The occurrence of subarachnoid hemorrhage is associated with increased mortality [1]. Septic embolism usually occurs in left-sided cardiac infections; however, in rare circumstances, right-sided cardiac infections can cause intracranial complications via right-to-left shunts, such as a patent foramen ovale (PFO), through a paradoxical septic embolism [2]. However, to our knowledge, subarachnoid hemorrhage resulting from a paradoxical septic embolism associated with right-sided cardiac infection has not been previously reported.

We report a rare case of subarachnoid hemorrhage caused by the rupture of an infectious intracranial aneurysm resulting from a presumed paradoxical septic embolism*.*

## Case presentation

An 85-year-old woman had undergone aortic valve replacement with a bioprosthetic valve for severe aortic regurgitation and permanent single-chamber pacemaker implantation for symptomatic bradycardia with prolonged pauses 7 years before presentation. Thereafter, she was maintained on aspirin at a dose of 100 mg/d. Three months before admission, she underwent tooth extraction for a dental root infection. In addition, she habitually consumed dairy products, including unheated processed cheese.

She presented with right lower extremity pain, followed by left upper extremity weakness of unknown onset. On arrival, her blood pressure was 107/75 mmHg, and she was afebrile. Physical and ophthalmological examinations revealed no peripheral stigmata of infective endocarditis. Neurological examination demonstrated mild weakness in the left upper extremity. Laboratory testing showed a mildly elevated C-reactive protein level of 4.1 mg/dL, whereas creatine phosphokinase and D-dimer levels were within normal ranges.

Computed tomography (CT) of the head revealed a small subarachnoid hemorrhage over the right frontal convexity, and contrast-enhanced CT angiography showed a 1.5 mm saccular aneurysm in the distal right middle cerebral artery (Fig. 1). Therefore, aspirin therapy was discontinued.

**Fig. 1:**
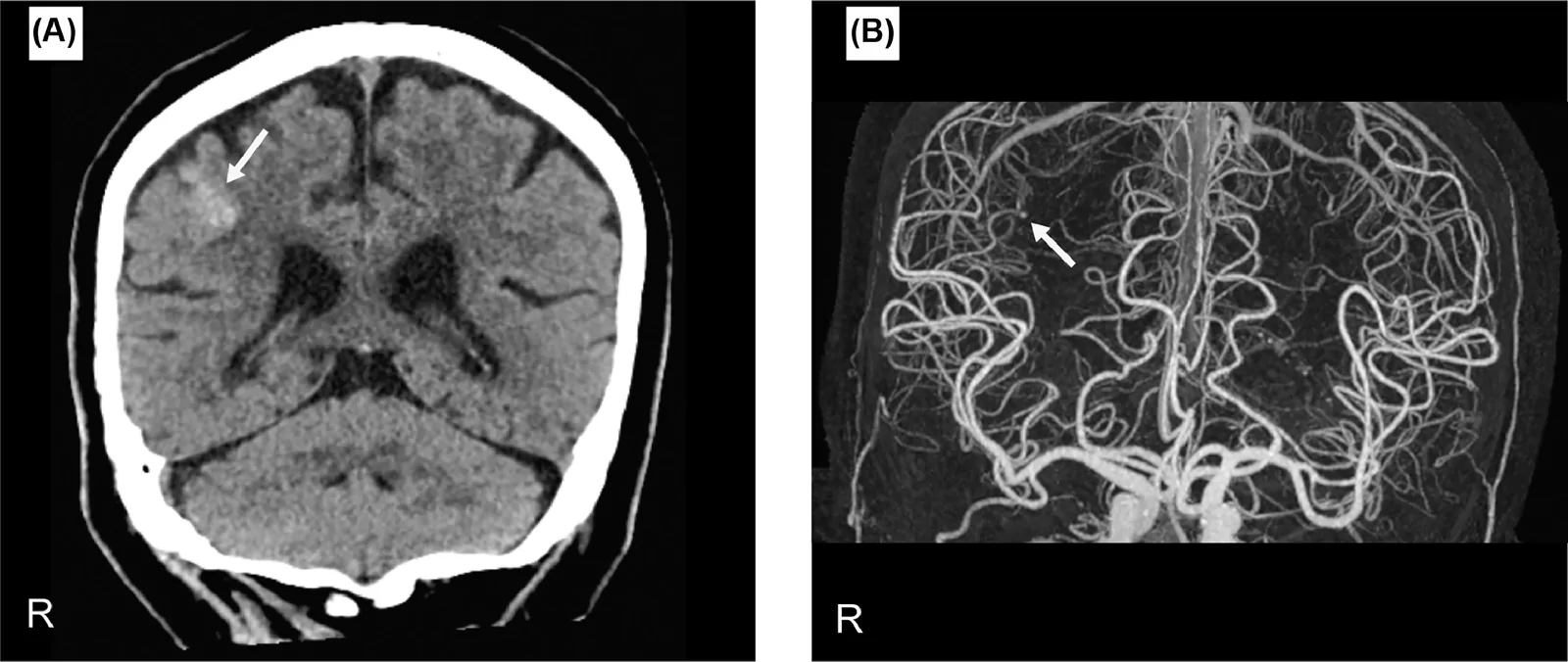
Imaging findings of convexity subarachnoid hemorrhage and distal infectious aneurysm. (A) Non-contrast head computed tomography demonstrates a small subarachnoid hemorrhage localized to the right frontal convexity (white arrow). (B) Contrast-enhanced head computed tomography angiography demonstrates a 1.5 mm aneurysm in the distal right middle cerebral artery (white arrow), consistent with a peripheral infectious aneurysm.

Transthoracic echocardiography revealed a mobile, 6 mm vegetation attached to the right ventricular pacemaker lead (Fig. 2A). No vegetation, abnormal structures, or increased flow velocities were observed in the prosthetic aortic valve. All three sets of blood cultures grew *Listeria monocytogenes,* and intravenous ampicillin (2 g every 4 hour) and gentamicin (80 mg every 8 hour) were administered. Device extraction and surgical treatment of the aneurysm were considered; however, these interventions were not pursued owing to the patient’s refusal and the high neurological risk.

**Fig. 2:**
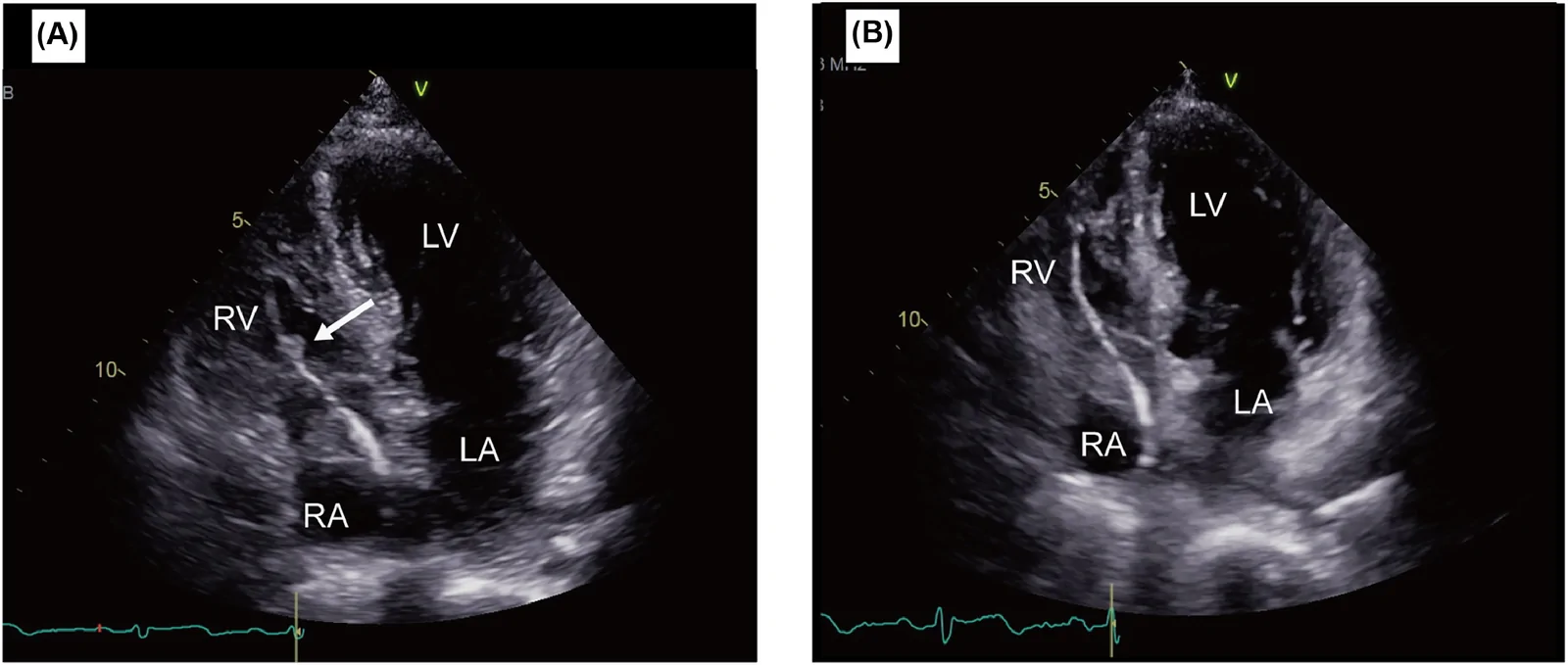
Transthoracic echocardiographic findings of pacemaker lead vegetation. (A) Transthoracic echocardiography shows a mobile vegetation measuring approximately 6 mm on the right ventricular pacemaker lead (white arrow), consistent with vegetation. (B) Follow-up transthoracic echocardiography on hospital day 32 demonstrates resolution of the vegetation after antibiotic therapy. Abbreviations: LA, left atrium; LV, left ventricle; RA, right atrium; RV, right ventricle.

Cerebrospinal fluid examination revealed no evidence of meningitis, and additional imaging studies identified no alternative infectious foci.

Repeat blood cultures obtained on day 8 of hospitalization were negative. Transesophageal echocardiography (on hospital day 11) and cardiac contrast-enhanced CT (on hospital day 16) revealed no evidence of infection involving the prosthetic aortic valve, left atrium, or left ventricle. However, a PFO with a right-to-left shunt (shunt grade 2) was identified.

Follow-up transthoracic echocardiography on day 32 of hospitalization demonstrated complete resolution of the vegetation on the right ventricular lead (Fig. 2B). Because the patient remained clinically stable with complete resolution of symptoms, additional cerebrovascular imaging of the aneurysm was not performed during follow-up.

Intravenous antibiotics were administered for 6 weeks, after which the therapy was switched to oral ampicillin (250 mg three times daily). The patient was discharged on hospital day 49.

## Discussion

From a radiological perspective, this case highlights the importance of multimodal imaging in identifying peripheral infectious aneurysms and their underlying etiology. This case represents an extremely rare instance of subarachnoid hemorrhage caused by the rupture of an infectious intracranial aneurysm, presumably resulting from a paradoxical septic embolism originating from vegetation on a right ventricular pacemaker lead. To the best of our knowledge, although cases of stroke and brain abscess caused by paradoxical septic embolism through a PFO have been reported, no previous reports have described infectious intracranial aneurysms or subarachnoid hemorrhages arising from this mechanism.

Neurological complications occur in approximately 25% of patients with infective endocarditis and include ischemic stroke, intracranial hemorrhage, and subarachnoid hemorrhage secondary to infectious intracranial aneurysms [3]. These cerebrovascular complications are typically associated with left-sided infective endocarditis and are considered rare in right-sided diseases. However, in the presence of a right-to-left shunt, such as PFO, septic emboli originating from the right heart may enter the systemic circulation and cause cerebral complications via paradoxical septic embolism [4].

In the present case, vegetation was identified exclusively on the right ventricular pacemaker lead, with no evidence of infection in the left cardiac chambers or bioprosthetic aortic valve. This finding supports paradoxical septic embolism as a plausible mechanism of infectious aneurysm formation and rupture, although the possibility of a subclinical prosthetic valve infection cannot be completely excluded. Although the patient was afebrile, the presence of a peripheral cerebral aneurysm, a characteristic of infectious aneurysms, and elevated inflammatory markers prompted early suspicion of infection and timely initiation of appropriate antibiotic therapy, likely contributing to favorable outcomes.

*Listeria monocytogenes* is a well-known cause of meningitis and perinatal infection, but is a rare cause of infective endocarditis or cardiac implantable electronic device infection. Dairy products, particularly ready-to-eat items such as unheated processed cheese, are recognized as important vehicles for *L. monocytogenes* transmission in the general population [5]. In this patient, habitual consumption of such dairy products may have contributed to gastrointestinal colonization, while poor oral hygiene and recent tooth extraction could have facilitated transient bacteremia and subsequent seeding of the pacemaker lead. Elderly patients are at an increased risk of invasive listeriosis, which may contribute to their susceptibility to *L. monocytogenes* infection.

In conclusion, right-sided device infections in patients with right-to-left shunts may lead to severe cerebrovascular complications through a paradoxical septic embolism. In cases of subarachnoid hemorrhage due to an infectious intracranial aneurysm, evaluation of a right-to-left shunt, including a PFO, is warranted.

## Declaration of generative AI and AI-assisted technologies in the writing process

During the preparation of this work, the authors used ChatGPT (OpenAI) to assist with language editing and improvement of clarity. After using this tool, the authors reviewed and edited the content as needed and take full responsibility for the content of the published article.

## Patient consent

Written informed consent was obtained from the patient for publication of this case report and accompanying images.

## Glossary

CT: Computed tomography
PFO: patent foramen ovale.
